# Early childhood development: the role of community based childcare centres in Malawi

**DOI:** 10.1186/2193-1801-3-305

**Published:** 2014-06-24

**Authors:** Alister C Munthali, Peter M Mvula, Lois Silo

**Affiliations:** Centre for Social Research, University of Malawi, Zomba, Malawi; Chancellor College, University of Malawi, Zomba, Malawi

**Keywords:** Malawi, Early Childhood and Development, Childcare

## Abstract

**Background:**

Somatic changes including growth and development of the brain of a human being occur very early in life. Programmes that enhance early childhood development (ECD) therefore should be part of the national agenda. Cognizant of this fact, the Malawi Government together with development partners facilitated the establishment of community-based child care centres (CBCCs) which are owned and managed by community members. This study was aimed at understanding how CBCCs operated and their core functions.

**Methods:**

Using information from databases kept by the District Social Welfare Officers from all the 28 districts in Malawi, coupled with snowballing, all functioning CBCCs were enumerated. A questionnaire was administered to the head of the CBCC or a care giver. Highly trained Research Assistants also carried our observations of the structures around the centres and the activities that actually happened. Data was analysed using a Statistical Package for Social Sciences.

**Results:**

Communities provide structures, support for care givers, food, utensils, labour and play materials for the children in CBCCs. The first ECD centre was established in 1966 but the real surge in establishing these happened towards the end of the 1990s and by 2007 there were 5,665 CBCCs in Malawi caring for 407,468 children aged between 3 and 5 years. CBCCs were established to provide pre-primary school learning, and in some cases provide special care to orphans and other vulnerable.

**Conclusions:**

Despite the fact that most CBCC premises and structures fell short of the standards laid down by the CBCC profile, the activities and services provided were mostly to the book. Children were provided with nutritious foods and subjected to play that stimulated their cognitive and mental development. Despite the fact that some members of the community do not realize the value of the CBCCs, the existence of these institutions is an opportunity for the community to take care of their children communally, a task that has become imperative as a result of the upsurge in the number of orphans as a result of the HIV and AIDS epidemic. The study recommends that Malawi should take investments in ECD programmes as a priority.

## Background

About 200 million children globally fail to reach their potential in cognitive development because of inter-related factors of poverty, inadequate care and poor health (Grantham-McGregor 2007). This is especially the case in resource poor countries such as Malawi. The high prevalence of poverty generally leads to inadequate food and poor hygiene and sanitation which consequently increase the vulnerability of children to diseases including malnutrition and related disorders. Severe clinical malnutrition also leads to deficits in intelligence and school performance. These issues affecting children need to be urgently addressed if developing countries are to achieve MDGs 1 and 2 which are to eradicate poverty and hunger; and to ensure that all children complete primary schooling, respectively. The first few years of human development are crucial as it is a time when somatic changes including growth and development of the brain occur (Walker et al. 2007).

In general early cognitive development is one of the major factors that determine school performance and progress at a later stage in life. There is a strong association between early childhood factors and primary school performance and progress: it has been demonstrated that children who have participated in early childhood education and related programmes generally remain in school, are unlikely to repeat classes and their class performance is much better compared to those who have never attended pre-primary programmes. In sub-Sahara Africa not many pupils complete their primary school and school retention is generally a major challenge. In Lesotho only 60% of the children who start school survive till Grade 7 (Department of Education, OSISA and UNESCO 2007). While Malawi has generally attained universal access to education, the country also experiences the challenge of school retention: only 30% of the children who start Standard 1 reach Standard 8, the last class in primary school before going into secondary school (Kadzamira et al. 2004). Within such a context where school retention is a challenge, increasing access to ECD programs would help in terms of creating interest in school and ensuring school retention among the school going age children.

Early school learning also improves children’s health and nutritional status. The proportion of children aged less than five years of age who are underweight declines as the preschool coverage in a country increases (Department of Education, OSISA and UNESCO 2007 and Garcia et al. 2007). It has also been demonstrated that stimulation and the provision of food supplements to improve children’s nutritional status improves motor and mental development and cognitive ability (Walker et al. 2007 and Engle et al. 2007). Early childhood programs also contribute towards reduction of fertility rates, for example Naidoo (2007) says that the incidence of motherhood for girls aged 10–18 is much less among those who had attended preschool as children compared to girls of the same age who had not attended preschool. The benefits of preschool exposure are well understood but only 12% of the preschool age children in Sub-Saharan Africa aged between 3 and 6 years are actually enrolled in preschool (Garcia et al. 2007). The benefits of ECD programs only appear 2–3 decades later in life (van der Gaag and Tan undated).

Since the benefits of ECD are well known, it is important that countries in the African region should aim at increasing the coverage of preschool education. Despite the evidence that ECD programs have significant positive impacts on children’s later life and on the wider society, investments in such programs especially in resource poor countries where there is a huge number of vulnerable children who would benefit from them the most have been low (see Irwin et al. undated). In Sub-Saharan Africa, less than 10% of public education expenditure goes to pre-primary education and this is also the case among donors (Department of Education, OSISA and UNESCO 2007). In their work in South America, van der Gaag and Tan (undated) argue that in general ECD programs are very expensive. It has been argued that in some countries in Africa publicly funded ECD constitute a relatively new concept and in countries where there are prevailing teething problems with delivery of primary school education, there is a need to address these problems first before embarking fully on ECD programs (Thomas and Thomas 2009). This article describes a nation-wide community-based and community-owned ECD program in Malawi facilitated by the Government of Malawi but run and managed by community members.

### Background information on community-based childcare centres in Malawi

Malawi is one of the countries with the most extensive network of ECD centres in Africa. These centres comprise of, among others, community-based childcare centres (CBCCs), pre-schools and day care centres (Yallow et al. 2012). These ECD centres were established in order to ensure that children in Malawi benefit from such interventions. The emphasis on ECD interventions such as the establishment and running of CBCCs is based on the evidence that exposing children to such programs ensures that they develop their basic skills, attitudes, behaviours and values that will last for their life time (Yallow et al. 2012). The CBCCs cater for children aged 3–5 years and are managed by members of the community namely parents, guardians, caregivers and community members (Messner and Levy 2012). The Ministry of Gender, Children and Social Welfare, with support from UNICEF, has been in the forefront in facilitating the establishment of CBCCs including the training of community volunteer caregivers who take care of the children enrolled in these centres. Apart from the Ministry of Gender, Children and Community Development, there are also some NGOs that are supporting CBCCs for example through the training of caregivers running these CBCCs, providing play materials and cooking utensils and the construction of CBCC structures. Each CBCC has a CBCC committee which consists of community members who oversee the daily functioning of the centre (Messner and Levy 2012).

Pre-school childcare is not a recent phenomenon in Malawi: as early as the 1950s some primary schools established by missionaries enrolled under-school age children for 2 years before they started Standard 1. It was only in 1966 that the first ECD centre was established by the Church of Central Africa Presbyterian in Blantyre, Southern Malawi, after which a number of other pre-school groups opened in Blantyre and other towns (Kalyati 2006). The commemoration of the International Year of the Child (IYC) in 1979 enhanced the opening of more pre-schools at every district centre in the country and by 1980 pre-schools were opened in all 24 districts of the country. Most of these pre-schools, however, had an urban presence. The CBCC programme was established in 1989 on a pilot scale in Mzimba, Salima and Chikwawa Districts with the aim of contributing to increasing access to ECD. The three pilots survived briefly due to communities’ minimal support and commitment. Towards the end of the 1990s Malawi witnessed an increase in the number of CBCCs due to the growing numbers of orphaned children as a result of the HIV and AIDS epidemic (Kalyati 2006). Currently, these community-owned and managed centres are all over the country and the majority are located in rural areas.

These CBCCs promote the holistic development of children through the provision of services such as essential health care, community integrated management of childhood illnesses, psychosocial care and support, water and sanitation, nutrition and stimulation and play. The promotion of holistic childhood development is also done through the building of capacity of the parents and caregivers in areas such as provision of psycho-social support, ECD, agri-business and business management. At CBCCs, children gather to access ECD services and are assured of at least one meal a day to support their nutritional needs.

### The study on CBCCs

While Government, development partners and other stakeholders appreciated the role that CBCCs were playing in the cognitive development of children, the exact number of these CBCCs remained unknown and so were the numbers of children enrolled in the CBCCs, the number of caregivers and members of CBCC committees who had been trained, the type and range of services that the CBCCs were offering to children and the type of infrastructure and equipment including play materials and cooking and eating utensils that were available in these CBCCs. This study was, therefore, conducted in order to create an inventory of CBCCs operating in Malawi in the years 2006–2007. Using data from this study, this descriptive paper explains how CBCCs are established; the community’s rationale for establishing the CBCCs; the extent to which the community is involved; the number of CBCCs operational in Malawi; the number and age of children cared for in these centres; the admission criteria; the type of services that the CBCCs are offering; and the problems encountered in providing these services. In addition the article also explores the type of infrastructure and equipment, including playing materials, available and sources of support, financial and otherwise since these are part a CBCC set-up. The paper further describes the role of the CBCCs in early childhood and development programs.

## Results

### Number of CBCCs in Malawi, their initiation and registration

Administratively Malawi is divided into three regions: the northern region has 6 districts and a total population of 1,708,930 (13.1%); the central region has 9 districts with a population of 5,510,195 (42.1%); and the southern region has 13 districts with a population of 5,858,035 (44.8%). This national survey found that there were 5,665 CBCCs in Malawi: 50.3% were in the southern region and 28.6% and 21.2% were in the central and northern regions, respectively. Eighty six per cent (86%) of the CBCCs operated 5 days a week and nearly all of them were open in the mornings only. Table 1 shows the year when the enumerated CBCCs were established.

**Table 1:** **Years when CBCCs were established by October 2007**

Year when CBCCs were established	Number of CBCCs established in that year
Before 1997	136
1998	72
1999	115
2000	255
2001	281
2002	379
2003	744
2004	765
2005	1140
2006	1169
2007	608

Of the CBCCs surveyed only 136 (2.4%) were established before 1997. Since 1998 the number of CBCCs has been increasing: the peak was in 2006 when a total of 1169 (20.6%) CBCCs were established. Ideally, the establishment of CBCCs is a community initiative. However, 45% of the respondents said that their CBCCs were initiated by Civil Society Organisations (CSOs), 42% by the local communities themselves, 5% by Government and 9% by other agencies which included City Assemblies and the University of Malawi. During in-depth interviews with caregivers, informants explained that while CSOs played an important role in the establishment of CBCCs, most CBCCs, however, initiated by communities and their leaders, especially village headmen. Once CBCCs have been established, they are supposed to be registered with the DSWO. Fifty six per cent (56%) 1of the respondents reported that their CBCCs were registered with the DSWO, 38% with CSOs, 1% with the City Assembly and 6% by other organizations. Overall 89% of the CBCCs were registered but it is evident that a significant proportion of the CBCCs were either not registered or they were registered with an inappropriate organization. This might be an indicator that there is generally a lack of knowledge about the organization or institution that the CBCCs are supposed to register with.

### Community’s rationale for establishing CBCCs

Most of the informants were aware that CBCCs were established in order to provide pre-primary school learning to children. Their view was that pre-primary school learning offered by CBCCs provides a strong educational foundation for children. They explained that CBCCs provide children with both learning and association skills; enable children to easily adapt to primary school environment; and, that such children perform very well in primary school compared to those who have not been enrolled in pre-schools. In some communities orphan care centres were established to provide basic necessities such as food, clothes, blankets and education to orphans and other vulnerable. Most informants added that some of the orphan care centres have, however, been transformed into CBCCs because of the demand for pre-school education among non-orphaned children and also as an attempt to prevent the discrimination of orphans. According to informants, the demand for pre-school education has also come about because of the presence of an increasing number of idle children in communities due to, among other factors, being very young for enrolment into primary school; lack of educational facilities within a reasonable walking distance; no one being at home to take care of children; and parents/guardians failure to pay a fee required at private nursery schools. Consequently, these idle children disturbed their parents and guardians from performing economically productive activities. Thus, the establishment of CBCCs was meant to preoccupy such children with certain activities and prevent them from disturbing guardians in doing their work. CBCCs were also established in order to provide protection to young children. Some respondents revealed that young children in most communities are vulnerable to many threats. Children are at risk of being bullied, physical and sexual abuses and accidents when they are left alone at home. Having a CBCC in a community therefore, meant that children were less exposed to such threats.

Finally, some CBCCs were established because community members admired CBCCs in other communities. They were impressed by the school performance of children from other CBCCs including their ability to communicate in English. In addition, communities also admired the donations that other CBCCs were receiving. Some communities were unhappy to see children from other communities patronize their CBCCs. Communities with no CBCCs were therefore being criticised by other communities with CBCCs for being dormant in that instead of establishing their own nursery schools, they just sent their children to other peoples’ schools. Communities with no CBCCs were therefore motivated to establish CBCCs within their community through receiving such criticisms. It is therefore evident that CBCCs were established for various reasons but most importantly to provide a learning environment for children within the community and create an opportunity where orphans and non-orphans can mix in a school environment.

### Admission criteria into CBCCs

Caregivers explained that age was the most important factor when enrolling children in CBCCs. Older children are considered eligible to start primary school education while those that are younger are considered difficult to handle. The CBCC profile recommends that CBCCs should admit and register children who are aged three to five years old (Ministry of Gender, Children and Community Development and UNICEF 2007). While this is the case, caregivers in CBCCs explained that there are always exceptions. For example, two year old children can also be enrolled if they are orphans or if their parents are too ill to take care of them. Children younger than 2 years are encouraged to stay at home so that they can be breastfed and enjoy early learning activities at home. During the survey children were grouped into three categories: less than three years; three to five; and over five years. This survey found that 65.1% of the children registered are within the 3–5 year age range as recommended by the CBCC profile; 26.8% were younger than 3 years; and 8.1% were older than 5 years. At national level, it is therefore evident that 92% of the children enrolled at the various CBCCs were aged less than 5 years. These results also tally with what was obtained in the in-depth interviews where most respondents reported that their CBCCs enrolled children between two and five years of age.

Almost all CBCCs enrol children regardless of their physical disability, social status and religious belief. Non-orphans constitute the largest proportion of children registered in most CBCCs. Children with special needs include the visually impaired, those who have speech impairment, malnourished children, children with epilepsy and those who are HIV+. Most CBCCs, however, indicated that they do not have children with special needs because of the absence of such children in their catchment area. Very few CBCCs indicated that they do not enrol extreme cases of children with special needs. For example a caregiver in Nsanje stated that

“*We do not enrol them because they give us tough time to manage them…*” (Anonymous caregiver, Nsanje District).

The lack of appropriate training and resources makes it hard for CBCCs to handle children with special needs. Caregivers also said that on enrolment they also consider the child’s ability to communicate. If a child fails to communicate, some CBCCs do not register him/her mainly because he or she may fail to interact well with his or her friends and caregivers. A child’s behaviour further determines whether or not to enrol him or her into the CBCC. Children who bring about problems within the CBCC are rejected in some CBCCs. Normally, the distance that a child is required to travel to the CBCC is also considered. In most CBCCs, children that live within a walking distance from the centre are the ones who are usually enrolled. Similarly, those coming from distant places are mostly denied enrolment. Finally, the commitment of the parents/guardians towards making contributions to the CBCC determines whether their children should be enrolled or not. This is especially the case because the food that children eat in the CBCCs is mostly contributed by parents and other community members.

### Number of children enrolled in CBCCs

A total of 407,468 children were registered in the CBCCs that were enumerated in 2006–2007. Of these children, 183,810 were boys and 223,658 were girls. As was the case at national level, in all the districts there were more girls than boys. Overall 21.9% of the children enrolled in CBCCs in Malawi were orphans while a smaller proportion (3.5%) were children with special needs. The study also found that 92% of the CBCCs nationwide kept some form of register which among other particulars showed the name of the child, attendance and reasons for absenteeism.

### Ownership of premises from which CBCCs operate

There are guidelines on the type of building and environment in which children can learn and develop safely and communities are encouraged to use whatever buildings may be available locally, provided they are safe for children. These structures include churches, old shops and individuals’ homes (Ministry of Gender, Children and Community Development and UNICEF, 2003). An ideal CBCC is fully supported by the community and it is supposed to have a sound, spacious, well lit and well ventilated building, a roofed play area, adequate latrines, bathrooms and a kitchen separated from the main building with a food store, among others. Ownership in this context meant the building was available and CBCC management did not have to ask for permission from anyone in order to use it. They were also responsible for maintaining the building. Overall, only 30% (1698) of the CBCCs enumerated reported having their own building while 70% (3967) did not – they used premises belonging to other organisations. Overall 43.5% of the CBCC structures were being used for other activities when not being used for CBCC activities. Table 2 below shows the other uses of CBCC buildings.

**Table 2:** **Other uses of CBCC premises**

*Other uses of CBCC buildings*	*Proportion of respondents who mentioned this*
Children’s corner	18.0
Prayers	25.2
Party meetings	7.9
School	12.9
Clinic	13.8
Seminars	13.1
Welfare committee meetings	37.9
Other uses	15.6

Table 2 generally shows that the CBCCs were mainly being used for other community initiatives such as welfare committee meetings (37.9%), prayers (25.2%) and children’s corners (18.0%). While the buildings were being used for other activities, it is evident that more than half of the buildings were not being used for any other activities. A good proportion of the CBCC structures were grass thatched with mud/earth floors and walls made of grass/reeds. This demonstrates that a good proportion of CBCCs were not permanent structures as recommended by the CBCC profile.

### Sources of funding and banking

While donors may fund the operations of CBCCs (5.1%), 87.2% of the CBCCs, however, reported that it is the community itself that funds the operations of the CBCCs. Almost all CBCCs depend on contributions from community members including parents and guardians of the children enrolled in the CBCC, CBCC committee members, caregivers and village headmen. At the time of the study parents or guardians of children attending CBCCs contributed money towards CBCCs and this ranged from K5 to K300 per month. These CBCCs serve poor communities who find it difficult making contributions; hence such contributions are inadequate. Some CBCCs also conduct fund raising activities such as doing *ganyu* (piece work), selling bricks and firewood, dairy farming, commercial bee keeping and video shows. These activities are conducted in order to raise money for the CBCC. The provision of external direct financial resources to CBCCs is rare; however donations such as teaching and learning materials; food; farm inputs; and medical resources (mosquito nets and drugs) are not uncommon. There are also some CBCCs which have received support for construction of their buildings.

### Sources of water for CBCCs

In terms of drinking water, 65% of the CBCCs enumerated drew their water from boreholes, 12% from unprotected wells, 11% from pipes, 8% from the rivers, 4% from protected wells and 1% from the house of the caregiver. Very few of the water sources, were within the CBCCs (15.2%) or at a distance of less than 100 metres (30%). Across all the districts, only 36% drew water from a distance of between 100 metres and less than 500 metres and 36% had their water source between 500 metres and less than one kilometre. Only 6% drew water from a distance of more than one Kilometre. Most of the CBCCs therefore were less than 500 metres from their source of drinking water.

### The provision of health services in CBCCs

There are times when children fall sick as they are attending classes at the CBCCs. The CBCC profile recommends that such children should be taken to their parents or guardians who can then seek treatment for the child (Ministry of Gender, Children and Community Development and UNICEF 2007). The majority of the caregivers (88%) indicated that they take the child to the parent or guardian; 20% indicated that they themselves take care of the child and 17% indicated that they take the child to the nearest health centre as can be seen in Table 3 below.

**Table 3:** **Action taken when child is sick at a CBCC**

*Action taken when child is sick*	*% of respondents who mention this*
Take the child to parent or guardian	88
Caregiver takes care	20
Home based care volunteer treats the child	4
HSA treats the child	2
Buy medicines from shops	13
Go with child to traditional healer	1.1
Take child to health centre	17
Never had a child who fell ill	2

Thirteen per cent (13%) indicated that they buy medicines from the shops and treat the child. The use of traditional medicines, home based care volunteers and HSAs in the treatment of children who fall ill at CBCCs is quite rare. The CBCC profile also recommends that CBCCs are supposed to have sick bays where children that are not feeling well could rest: however this survey found that only 8.4% of CBCCs had such a facility. Thirty seven per cent (37%) of the CBCCs reported being visited by health personnel. During such visits health workers are involved in the provision of several services such as conducting health education talks (61%), promoting hygiene and sanitation (65%), Vitamin A supplementation (50%), growth monitoring and health promotion (47%), immunization (40%), de-worming (34%) and HIV/AIDS awareness campaigns (15%). While just more than a third of the CBCCs enumerated were visited by a health worker, it is evident that the services they provide are important childhood interventions.

### Nutrition in CBCCS

Providing nutritious food or snacks once or twice a day for the children in its care is an important part of a well-run CBCC and indeed any ECD centre. At national level only 18.6% of the CBCCs had a kitchen where they prepared food for the children. Overall 91.2% of the enumerated CBCCs provided meals for children. CBCC management confirmed that if food is not present in the CBCC children’s attendance rate significantly goes down. Table 4 below shows the type of food that was being provided by CBCCs.

**Table 4:** **Type of food provided by CBCCs**

*Type of food provided by CBCCs*	*% of CBCCs that provided the food*
Maize flour porridge	77
Soy porridge	32
Tea without milk	17
*Likuni phala*	12
*Nsima* with vegetables	9
Porridge with groundnuts/margarine	9
Tea with milk	7
Rice with meat, beans and vegetables	3
Other means	22

Porridge prepared using maize flour was the most common food provided by the CBCCs followed by soy porridge. More nutritious foods such as *nsima* with beans and vegetables and rice with meat/beans are rarely provided. A small proportion of CBCCs provided tea, but usually without milk. In general there was a lack of eating and cooking utensils within CBCCs. Only 41% had cooking pots; 39% had plates; 36% had cups while 32% had spoons. In terms of sources of food, most of this was sourced within the community as can be seen in Table 5 below.

**Table 5:** **Sources of food for CBCCs**

*Sources of food*	*Percentage*
Parents	60
Well-wishers	26
Communal gardens	30
Purchasing food	14
Community members	38
Other sources	24

Parents of children enrolled in CBCCs were the major sources of food that children ate. This was followed by committee members, CBCCs growing their own food and then obtaining food from well-wishers. It is therefore evident that most of the food that is used in the CBCCs is sourced from within the community. Communal gardens are an important source of food. Members of the community work in these gardens and the crops harvested are used to provide meals to the children enrolled in the CBCCs. Seventy five per cent (75%) of the CBCCs that owned communal gardens grew maize. Other crops included soya beans (25%), vegetables (16%) and groundnuts (14%). For those CBCCs which reported having a communal garden, parents of children attending CBCCs were major source of farm inputs (53%) followed by NGOs/FBOs (31%). The DSWO made a very small contribution in terms of farm inputs at 3%. These results generally demonstrate that CBCCs provide nutritious food to children and most of this food is sourced from within the community with minimal support from outside.

### Play and recreation in CBCCs

Play and social interaction are very important parts of ECD programs hence CBCCs should have enough in-door and out-door play materials to allow children a wide choice of activities. CBCCs reported a variety of activities that children did everyday as shown in Table 6 below.

**Table 6:** **Common daily activities done by children in CBCCs**

*Type of activity*	*% of CBCCs that have this activity*
Painting and drawing	54
Pasting	13
Clay modelling	74
Singing	99
Story telling	90
Puzzling	12
Free play	94
Rope skipping	67
Sand and water	23
Spiritual activities	90
Other activities	34

Singing was the most popular daily activity done by children in the CBCCs followed by free play, spiritual activities and story telling, clay modelling and then rope skitting. Other activities such as painting/drawing and pasting and puzzling were not common in the CBCCs. In terms of indoor and outdoor play materials, it was evident that CBCCs are not well equipped with in-door play materials. Forty percent (40%) of the CBCCs reported having soft balls and nearly a fifth of the CBCCs reported having picture and story books, art materials and assorted toys. Most of the play materials were reported by only 10% of the CBCCs surveyed.

## Discussion

This study has shown that in 2006/2007 there were just over 400,000 children enrolled in the 5,665 CBCCs enumerated throughout the country. UNICEF estimated that in 2011 a total of 771,000 children aged 3–5 accessed CBCCs and a further 187,500 accessed children’s corners (UNICEF 2011). This shows that over the period 2006–2011 there was a significant increase in the number of children attending CBCCs in Malawi. According to the National Statistical Office, the 2007 population projection for under-five children in Malawi was 2,579,724 and Malawi’s population in the same year was projected at 13,187,632 (National Statistical Office 2009). Nearly 20% of Malawi’s population consists of children under the age of 5 years. What we see therefore is that 15.8% of the total number of under-five children in Malawi is being catered for in the CBCCs. The northern region had the highest proportion of children enrolled in CBCCs at 22.4% followed by the southern region at 18.1% and then the central region at 15.8%. This demonstrates that with increased investment in CBCCs and ensuring that they are operational, a greater proportion of children can be served in CBCCs. CBCCs are spread across Malawi and are managed by members of the community hence providing the early childhood education that children need.

This study also generally shows that CBCCs, as ECD centres, are playing an important role in Malawi despite the challenges that are being experienced in the management of these centres. Even though no studies have been done to determine the impact of the CBCCs in the later life of the children who attend these centres, the Government of Malawi supports the centres based on results from other countries which have demonstrated that ECD programs prepare young children for enrolment in primary school and that many of the benefits of ECD are realised through improved enrolment and schooling achievement of ECD graduates (van der Gaag and Tan undated; Ministry of Gender, Children and Community Development and UNICEF, 2009) as well as school achievement (van der Gaag and Tan undated). The reasons for promoting ECD programs as found by van der Gaag and Tan were also mentioned by caregivers in this study who said that community members established CBCCs in order to expose these children to education as well as ensuring that when the children start Standard 1 they perform better in classrooms. Even though some authors have argued that early childhood care and education (ECCE) should not become an integral part of the education system and policy until resource poor countries can adequately sustain primary and basic schools (Thomas and Thomas 2009), in this study we have demonstrated that using community-based and community-owned approaches such as CBCCs costs of running such a system can be decreased significantly as everything is being run and managed by community members for example they provide meals given to enrolled children and they engage in fundraising activities to raise money for managing CBCCs. The major challenge, however, is the training of caregivers. Such training is the responsibility of a national core team of trainers (Messner and Levy 2012) and it is not cheap.

Van der Gaag and Tan (undated) have also argued that benefits of ECD programs are not only related to education and that there are also other direct benefits to the child. One of the examples they give is the provision of meals in ECD centres. The CBCCs in Malawi also provide at least a meal a day to the children enrolled in these centres; hence children are assured of at least one meal a day. In Malawi the prevalence of malnutrition has not changed much over the last 20 years: stunting among under-five children remains high at about 50% (National Statistical Office 2011; National Statistical Office and UNICEF 2008). The provision of meals therefore ensures that children enrolled in these CBCCs have at least a meal a day which contributes to fighting malnutrition in a country where this is quite prevalent. The provision of these meals in the CBCCs is also a major attraction for children and Munthali et al. (2008) found that if the CBCCs run out of food children’s attendance was low and such centres eventually closed.

In addition to the provision of nutritious foods the CBCCs also provide health care services for children as is the case with other ECDs (for example see van der Gaag and Tan undated). While it is recommended that when children enrolled in CBCCs are ill their parents or guardians should be informed so that they can take their children to the hospital (CBCC profile), health workers especially Health Surveillance Assistants (HSAs) do visit some CBCCs to provide services such as immunisation and other preventive and promotive health services. HSAs are the lowest cadre in the Ministry of Health in Malawi and are based at community level, attached to a health facility and mainly perform preventive and promotive health services (Ministry of Health 2011). Studies have also demonstrated that even though the GoM designed an Essential Health Package (EHP) which should be available to everyone, orphans and other vulnerable children have problems in accessing health care. For example, orphaned children aged 0–4 are less likely to have a health card and are also less likely to have received the required immunisation and Vitamin A supplements (Pullum 2008a, b). As has been reported in this study, a number of CBCCs did report that their facilities were visited by health workers providing various health interventions.

This study has also highlighted the fact that the enrolment of children in CBCCs tends to free up time for mothers or guardians of these children and hence they are able to perform other economically productive activities. A number of scholars have also highlighted the fact that ECD programs create or improve the employment opportunities of mothers hence they can earn an income (Yallow et al. 2012; van der Gaag and Tan undated; Messner and Levy 2012). In the current study informants also said that in general children are at risk of being bullied and they can experience physical as well as sexual abuse when they are left home and they did acknowledge that CBCCs are helpful because they offered protection to children against such threats. Yallow et al. (2012) also found that instead of being left alone in the home while their mothers or guardians go to work, the CBCCs provided considerable opportunities for protecting children against violence, abuse, exploitation and neglect (see Yallow et al. 2012).

What we see therefore is that just like any other approaches in ECD programming the CBCCs provide a combination of nutrition, health care, protection and cognitive development (see Young, 1996). There is a general consensus that ECD programs are particularly beneficial for disadvantaged children as most of the components that contribute to the proper development of a child are usually present in a relatively well-off household (van der Gaag and Tan undated). As has been demonstrated in this study overall 21.9% of the children enrolled in the CBCCs in Malawi are orphans while a smaller proportion at 3.5% are children with special needs. In Malawi there are 1.16 million orphans of which about 0.44 million are due to AIDS (National AIDS Commission 2012). While the extended family system is still a major approach for caring for orphans, this system is becoming overstretched (Ministry of Women, Children and Community Development 2010). Since poverty is widespread, members of the extended family system already living in poverty are further pulled down below the subsistence levels by an influx of orphans. In such prevailing poverty situations, it becomes difficult for the extended family system to adequately provide for the survival, protection and development of orphans. Because of poverty it is difficult for OVC to have a decent life: they lack parental care, healthcare, adequate shelter and good and nutritious foods. Furthermore, orphans are deprived of their right to education as detailed in the Constitution of the Republic of Malawi, the CRC and other international instruments. They will drop out of school even if school fees are paid for them (Ministry of Women, Children and Community Development 2010). Other vulnerable children such as street children and the disabled are also at risk of mistreatment and exploitation through child labour, sexual or physical abuse, malnutrition, child trafficking, and witchcraft practices. The establishment of CBCCs therefore is making a significant contribution as it is contributing towards ensuring children, especially that orphans and other vulnerable children, have access to nutritious foods, health care and psycho-social support. The enrolment of these orphans in the CBCCs constitutes an important component of the national response to the HIV and AIDS epidemic in Malawi and such an approach ensures that these vulnerable children have access to food, health care and protection. Messner and Levy (2012) also found that CBCCs constitute a major Government of Malawi endorsed response to the OVC situation especially those living in local communities. This study has also shown that there was an increase in the number of CBCCs in the 1990s and early 2000s. A number of studies (see Kalyati 2009 & Yallow et al. 2012) have highlighted the significant increases in the number of CBCCs and this was mainly to support the growing numbers of children left orphaned mainly due to the HIV and AIDS epidemic.

The sustainability of the CBCC approach to ECD programming lies in the fact that it is a community-owned and managed initiative. The community provides almost all the food that is eaten in these centres as has been demonstrated in this study. While the structures can be owned by the CBCC itself, it can also operate from borrowed buildings such as churches, in the open air or in grass thatched structures as shown in this study. There are a number of NGOs that have supported the construction of the CBCC structures (Messner and Levy 2012). The current study has also demonstrated that some of the CBCC structures are also used for other purposes if they are not being used by the CBCC. It has been argued that it is important to invest in ECD programs as it is a major mechanism through which intergenerational cycle of poverty can be broken (van der Gaag and Tan undated). This would therefore help resource poor countries such as Malawi to achieve MDG 1 target of reducing poverty levels.

## Conclusion

This study has shown that CBCCs, as ECD structures, which are established and managed by communities are playing an important role in communities by providing a pre-primary school learning environment and in 2006/2007 over 400,000 children were attending these CBCCs. While Malawi specific studies have not been done to determine the impact of CBCCs on the future development of the child, it is evident that these CBCCs ensure that children have access to pre-school education, health services and nutrition. In addition to this, the CBCCs admit a considerable proportion of orphaned children hence they constitute an important component of the national response to the HIV and AIDS epidemic. Apart from the training of caregivers which is the responsibility of the national authorities, all other activities within CBCCs are being managed by community members; hence such a community-based and a community-owned approach to ECD programming can be sustainable as long as communities are mobilised to support and own the process. There is a need for the Governments of Malawi to invest in ECD in order to meet the MDG for poverty reduction, education and health.

## Methods

Malawi is divided into three administrative regions namely the northern, central and southern regions. It has a total of 28 districts and this study was conducted in all these districts. In each district the District Social Welfare Offices (DSWOs) were the point of entry for the study team: they provided information on the number and location of the CBCCs that they knew in their districts. They have this knowledge because all CBCCs are supposed to be registered by their offices. The DSWOs were also requested to provide a list of their partners at district level who are involved in or support the operations of CBCCs. These partners provided information on the names and location of these centres. Once a list of all CBCCs was compiled at district level using information from the DSWO and other partners, all the CBCCs were visited. In addition to using the DSWO and other partners at district level, a snowball approach was also used to identify other CBCCs in each district. Snowballing involves informants referring the researcher to other informants, who are then contacted by the researcher. These informants in turn refer the researcher to yet other informants, and so on (Noy 2008). For each CBCC visited, caregivers were asked if they knew any other CBCCs in their area.

These approaches, namely using the DSWO (and its district partners) and snowballing, proved very useful in the identification of the CBCCs in the country. In addition to this, the research team used Health Surveillance Assistants (HSAs), community development facilitators, chiefs and community members to identify or confirm the existence of the CBCCs. HSAs constitute the lowest cadre in the Ministry of Health based at community level and responsible for approximately 1,000 persons and are mainly responsible for promotive and preventive health in their respective catchment areas (Ministry of Health 2011).

Community Based Organizations (CBOs) also proved very useful in providing this information as some CBCCs were reportedly established and were owned by CBOs and were being supervised by them. A questionnaire was administered to all CBCCs which were operational at the time of the survey. In addition, in-depth interviews were also conducted with chairpersons or their representatives at some of the CBCCs.

## Abbreviations

AIDS: Acquired immunodeficiency syndrome
CBCC: Community-Based Child Care Centre
CRC: Convention on the rights of the child
CSOs: Civil society organizations
DSWO: District social welfare officer
ECD: Early child development
EHP: Essential Health package
FBO: Faith-Based organization
HIV: Human immunodeficiency virus
HAS: Health surveillance assistance
MDGs: Millennium development goals
NGO: Non-governmental organization
OSISA: Open society initiative for Southern Africa
OVC: Orphans and vulnerable children
UNESCO: United Nations Educational, Scientific and Cultural Organization
UN: United nations
UNICEF: United Nations Children’s Fund.
